# Measuring nanoparticles shape by structured illumination

**DOI:** 10.1038/s41598-024-53665-1

**Published:** 2024-03-04

**Authors:** Shubham Dawda, Zhean Shen, Aristide Dogariu

**Affiliations:** CREOL, The College of Optics and Photonics, 4304 Scorpius Street, Orlando, FL 32816 USA

**Keywords:** Materials science, Optics and photonics

## Abstract

Exploiting the size and shape of nanoparticles is critical for engineering the optical and mechanical properties of nanoparticle systems that are ubiquitous in everyday life. However, accurate determination of nanoparticle morphology usually requires elaborated methods such as XRD or TEM, which are not suitable for non-invasive and rapid control. Dynamic light scattering on the other hand, relies on the motion of nanoparticles and mixes different rotational and translational diffusion coefficients to infer synthetic information about the shape in terms of effective hydrodynamic characteristics. Here, we introduce a new scattering approach for measuring shape. We demonstrate analytically, numerically, and experimentally that the contrast of low-intensity fluctuations arising from the scattering of classically entangled optical fields allows determining the polarimetric anisotropy of nanoparticles. By leveraging the active variation of illumination structuring, we control the non-Gaussian statistics of the measured fluctuations, which, in turn, provides means to improve the measurement sensitivity. This technique offers practical opportunities for applications ranging from molecular chemistry to drug delivery to nanostructures synthesis where the real-time, quantitative assessment of nanoparticles shapes is indispensable.

## Introduction

When imaging is inadequate, scattering based techniques are the methods of choice for determining the hydrodynamic sizes of small particles in colloidal dispersions. Nevertheless, finding the exact shape of submicron particles is still a challenge. This requirement becomes more and more stringent not only in the context of naturally occurring scattering media^1–4^ but also for characterizing the outcomes of a variety of nanotechnology enabled processes ranging from biomedicine^5,6^ to engineered coatings^7,8^ to nanoparticle synthesis^9^.

Characterizing non-spherical particles introduces new challenges especially when they are randomly oriented. However, even in such circumstances, shape information can still be retrieved by examining vectorial features of the fluctuations of scattered intensities^10–17^. For instance, when fluctuations are due to the random orientations of a single dipolar particle, one could employ stochastic scattering polarimetry to recover the polarizability tensor^12,13^. At the other extreme, when a sufficiently large group of independent particles generates a scattered field with complex Gaussian statistics^18,19^, techniques such as depolarized DLS^14,15^ and polarization fluctuation spectroscopy^16,17^ may be employed. While under intense scrutiny^17,20–24^, these techniques are rather limited by the intricate relation between rotational and translational diffusion coefficients and also by the stringent signal to noise requirements in the detection of orthogonally polarized fluctuations^25–27^. In many practical situations, however, one cannot examine one particle at the time, or, on the other hand, one cannot observe a very large number of particles such that the Gaussian statistics can be invoked. In such regimes, intensity fluctuations are due not only to the random displacements and orientations, but also to the random number of particles in the interaction volume. In this case, the intensity of scattered light is a non-gaussian random variable^19^. In the following, we will demonstrate a method that operates in such a low particle concentration regime^28^.

Most sensing approaches based on light scattering rely on a single, homogeneous interaction volume. We will show that by structuring the illumination one can create a plurality of interaction volumes from which statistically different fluctuations can be measured and processed to recover the shape anisotropy of the scattering particles. More precisely, we will consider a low concentration of monodisperse, randomly moving and orienting sub-wavelength anisotropic but axisymmetric particles. This ensemble is illuminated such that the interaction volume is divided into two spatially non-overlapping orthogonally polarized interaction volumes. We will demonstrate that by measuring just the contrast of intensity fluctuations, the optical aspect ratio of the particles can be retrieved. Not only can this be done without knowing the number of particles in the interaction volume, but this number itself can also be estimated.

We will first present a random-walk based model for the first two moments of intensity fluctuations arising from $M$ independent interaction volumes and discuss a procedure for adjusting the sensitivity for measuring optical aspect ratios of scattering particles. We will then illustrate a proof-of-concept demonstration of using cylindrical vector beams to generate a structured interaction volume from where the aspect ratio of low-concentration nanorods can be accurately measured.

## Dynamic scattering with structured illumination

**Figure 1:**
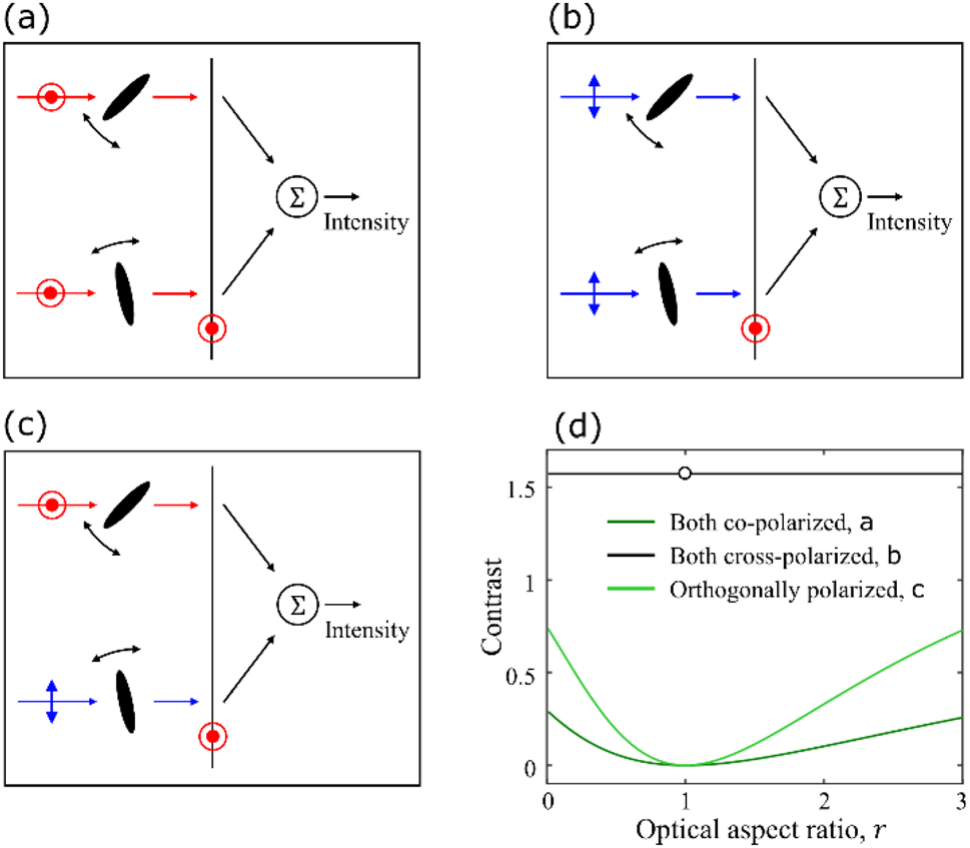
Contrast of intensity fluctuations as a function of optical aspect ratio for three types of structured excitations. Two randomly orienting anisotropic dipoles are excited by polarization states (**a**) parallel and (**b**) perpendicular to the analyzer and (**c**) excited by orthogonally polarized states. The contrast of resultant intensity fluctuations as a function of the optical aspect ratio is plotted in (**d**).

Let us first consider the canonical example of two anisotropic but axisymmetric dipoles fixed in space and uniformly randomly and independently orienting. Each dipole has a polarizability tensor $\overline{\alpha } = {\text{diag}}\left( {\alpha_{1} ,\alpha_{2} ,\alpha_{2} } \right)$ and an optical aspect ratio $r = \alpha_{1} /\alpha_{2}$. We compare the contrast of fluctuations of intensity scattered perpendicular to the direction of excitation as a function of $r$ under three conditions: when both dipoles are coherently excited by the same co and cross-polarized states and when they are excited by orthogonal states. This situation is schematically depicted in Fig. 1a–c. Using the scattered fields derived in ^29^, in supplementary section I, we derive the contrast of intensity fluctuations as a function of $r$. These dependences are plotted in Fig. 1d and, as can be seen, for identical excitations, the contrast is sensitive only when both excitations are polarized parallel to the analyzer. However, the sensitivity is higher when the excitations are orthogonal to each other. To intuitively understand this, we note that, according to supplementary equation (S1), the scattered field amplitude in a co-polarized geometry has a dipole orientation independent background term that does not exist in the cross-polarized geometry. This causes the orientationally averaged scattered intensity to be lower when both dipoles are excited in a cross-polarized configuration compared to when they’re excited in a co-polarized one, resulting in a higher contrast for the case in Fig. 1b than Fig. 1a. Since the case considered in Fig. 1c is an intermediate between the other two, the value of intensity fluctuations’ contrast also lies between the other two cases, resulting in a higher sensitivity of the contrast to the optical anisotropy. This suggests that if the excitation is structured such that different independently orienting dipoles are excited by orthogonally polarized fields, the sensitivity of the contrast to their optical aspect ratio may be improved.

Let us now examine the case of a group of independent particles. The particle concentration is low, and they act in single scattering regime such that the total scattered field can be considered as the coherent sum of the fields scattered by the individual particles. When the interaction volume is structured into $M$ independent interaction sub-volumes, the total scattered intensity ($I$) can be written as

1 $$I = \left| {\mathop \sum \limits_{m = 1}^{M} A_{m} \exp \left( {i{\Phi }_{m} } \right)} \right|^{2} = \left| {\mathop \sum \limits_{m = 1}^{M} \left( {\mathop \sum \limits_{n = 1}^{{N_{m} }} a_{n,m} \exp \left( {i\phi_{n,m} } \right)} \right)} \right|^{2} .$$

Here, $a_{n,m}$ is the scattered field magnitude and $\phi_{n,m}$ is the propagation phase of the field scattered by the $n{\text{th}}$ particle in the $m{\text{th}}$ sub-volume. Hence, $A_{m}$ and ${\Phi }_{m}$ are the magnitude and phase of the phasor corresponding to the fields scattered by particles in the $m{\text{th}}$ interaction volume. In a random walk interpretation^16,18,19,28–30^, Eq. (1) represents the resultant of the coherent superposition of independent random walks, each associated to a specific interaction sub-volume.

Assuming that (1) all ${\Phi }_{m}$ s are uniformly randomly distributed over a range of $2\pi$ radians, (2) $A_{j}$ and $A_{k}$ are independent random variables for $j \ne k$ and (3) $A_{j}$ and ${\Phi }_{j}$ are independent random variables, in supplementary section II, we derive the average and variance of $I$ as– $\left\langle I \right\rangle = \mathop \sum \limits_{m = 1}^{M} \left\langle {I_{m} } \right\rangle$ and $Var\left( I \right) = \left\langle I \right\rangle^{2} + \mathop \sum \limits_{m = 1}^{M} \left\{ {\left\langle {I_{m}^{2} } \right\rangle - 2\left\langle {I_{m} } \right\rangle^{2} } \right\}$ where $I_{m} = A_{m}^{2}$ is the scattered intensity from the $m{\text{th}}$ sub-volume and $\left\langle \ldots \right\rangle$ denotes an ensemble average over independent realizations of the particle group.

### Case of two scattering volumes

**Figure 2:**
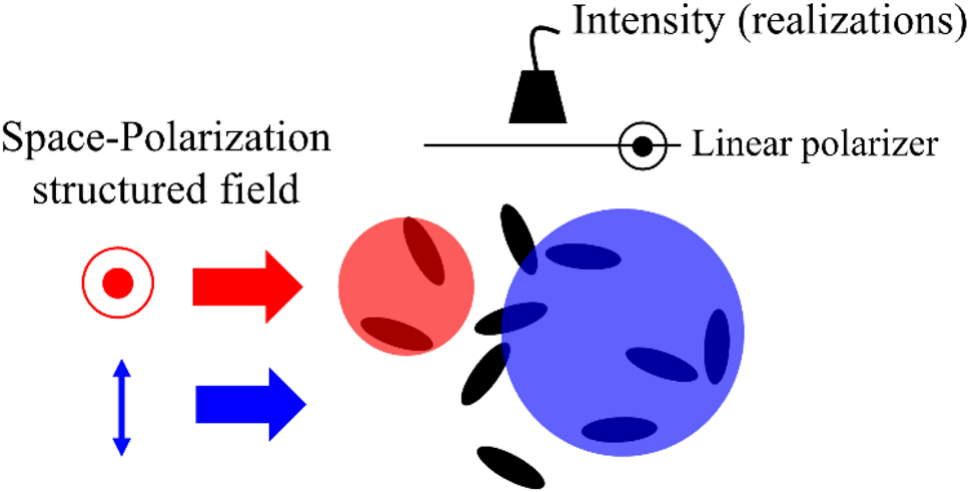
Space-polarization structured illumination. A space-polarization structured field illuminates a group of randomly translating and randomly orienting anisotropic particles creating two independent interaction volumes. The resultant intensity fluctuations are measured through a polarization analyzer.

We will illustrate this general model for the specific case of two different scattering volumes as depicted in Fig. 2. A space-polarization structured field illuminates a monodisperse group of axisymmetric sub-wavelength particles to create $M = 2$ homogeneously intense hard-edged independent interaction volumes. The scattered intensity polarized along one of the directions of incident polarization is measured perpendicular to the direction of illumination. In this case, the total interaction volume can be described by four parameters—the physical volume occupied by the co and cross polarized light interaction volumes, $V_{\parallel }$ and $V_{ \times }$, and their field magnitudes, $\left| {E_{\parallel } } \right|$ and $\left| {E_{ \times } } \right|$. With these parameters, we can define the structure of the total interaction volume by a volume ratio, $\nu = \frac{{V_{\parallel } }}{{V_{\parallel } + V_{ \times } }}$, and a field magnitude ratio, $E_{R} = \frac{{\left| {E_{\parallel } } \right|}}{{\left| {E_{ \times } } \right|}}$.

**Figure 3:**
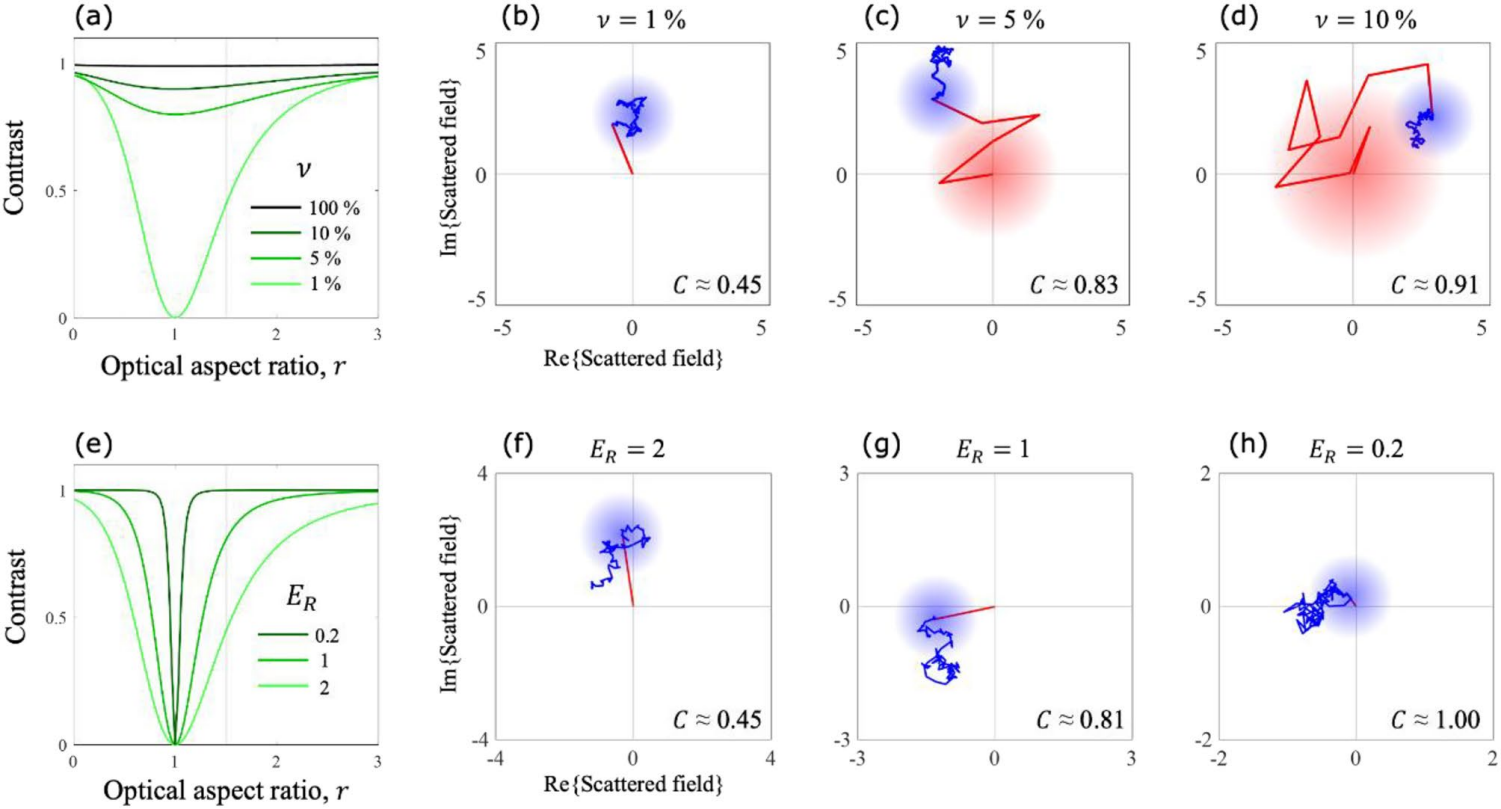
Constant number of particles in the interaction volume. (**a**) $C({\text{r}};{\upnu },{\text{E}}_{{\text{R}}} = 2)$ for a group of 100 particles. ${\upnu } = 100\%$ represents an unstructured interaction volume. Typical phasor diagrams of the scattered field for $r = 1.5$ and $\nu =$(**b**) 1%, (**c**) 5% and (**d**) 10%. (**e**) $C(r;\nu = 1\% ,E_{R})$ for a group of 100 particles. Typical phasor diagrams of the scattered field for $r = 1.5$ and $E_{R} =$(**f**) 2, (**g**) 1 and (**h**) 0.2.

For randomly translating and orienting particles, we use expressions of mean and variance derived above and those for moments of the resultant phasor in a random-walk^19^ to derive the contrast of intensity fluctuations, $C\left( {r,\left\langle N \right\rangle ,\left\langle {N^{2} } \right\rangle ;\nu ,E_{R} } \right) = \frac{Var\left( I \right)}{{\left\langle I \right\rangle^{2} }}$. The explicit expression is given in supplementary equation (S8). For static randomness, i.e. when the number of particles in the interaction volume is constant, the probability mass function of the number of particles in the total interaction volume will be $p_{N} \left( N \right) = \delta \left( {N - N_{0} } \right)$. In supplementary section III, we derive the contrast as

2 $$C\left( {r,\left\langle N \right\rangle = N_{0} ;\nu ,E_{R} } \right) = 1 + \frac{{f\left( {r;\nu ,E_{R} } \right)}}{{N_{0} }} - \frac{{g\left( {r;\nu ,E_{R} } \right)}}{{N_{0} }}$$

where expressions for $f\left( {r;\nu ,E_{R} } \right)$ and $g\left( {r;\nu ,E_{R} } \right)$ are derived in supplementary equation (S9). In Fig. 3a, the contrast $C\left( {r;\nu } \right)$ is plotted for $N_{0}$ = 100 particles and $E_{R} = 2$. The particles are distributed such that $\nu \cdot 100$ are in the co-polarized and the rest are in the cross-polarized volume. By comparing two equal interaction volumes, structured ($\nu \ne 100\%$) and unstructured ($\nu = 100 \%$), we show that the contrast is more sensitive to $r$ for a structured interaction volume.

In Fig. 3a, the point with $r =$ 1 and $\nu =$ 1% corresponds to the case of a single spherical dipolar particle in the co-polarized volume and 99 of those in the cross-polarized volume. Since there is no cross-polarized scattering from isotropic dipoles, the total scattered intensity results from a single isotropic dipole which scatters the same intensity for all positions within the interaction volume. Consequently, the intensity remains constant for all realizations and the contrast is 0. As $\nu$ increases, there is scattering from more and more isotropic dipoles and the intensity fluctuates because of their random positions. Hence, the contrast also increases.

To better understand the origin of sensitivity to $r$, we also plot the phasor diagram of the scattered field from a typical realization of the random particle group for $r =$ 1.5 while increasing $\nu$. In Fig. 3b–d, the red and blue phasors represent scattered fields from particles in the co and cross-polarized volumes, respectively. Under equal intensity excitations, the orientation averaged scattered intensity from an anisotropic dipole in the co-polarized volume is greater than that in the cross-polarized volume. In other words, the scattering from the co-polarized volume acts as a fluctuating background for the scattering from the cross-polarized volume. Since for $\nu =$ 1% (b), the background is the field scattered by a single dipole, it does not fluctuate due to the random positions and, consequently, the contrast has the smallest value. As $\nu$ increases, the background fluctuates due to both random orientations and positions of particles in the co-polarized volume, thus increasing the contrast.

For $\nu =$ 1% and $N_{0} =$ 100 particles, the variation of $C(r;E_{R})$ is illustrated in Fig. 3e. Note that the width of the “dip”, which is equivalent to the range over which the contrast is sensitive to $r$, can be varied by adjusting $E_{R}$. The difference in orientation averaged scattered intensities from an anisotropic dipole in a co and cross-polarized volume is maximized at $r = 1$ and decreases as $\left| {r - 1} \right|$ increases. As the difference decreases, the similarity in the scattered intensities from both interaction volumes increases, making the total scattering effectively be the same as that from a single interaction volume and contrast $\to 1$. However, this difference can be decreased or even increased by adjusting the ratio of excitation field magnitudes for both interaction volumes, $E_{R}$. We plot the phasor diagrams for a typical realization of the scattered field from a group of 100 particles with a decreasing $E_{R}$ (and hence decreasing difference of orientation averaged scattered intensities from a single dipole) in Fig. 3f–h where the contrast $\to$ 1 as $E_{R}$ decreases. Hence, from Fig. 3a and e, we also show that the sensitivity of the contrast to $r$ can also be adjusted by appropriately structuring the interaction volume.

### Role of number fluctuations

**Figure 4:**
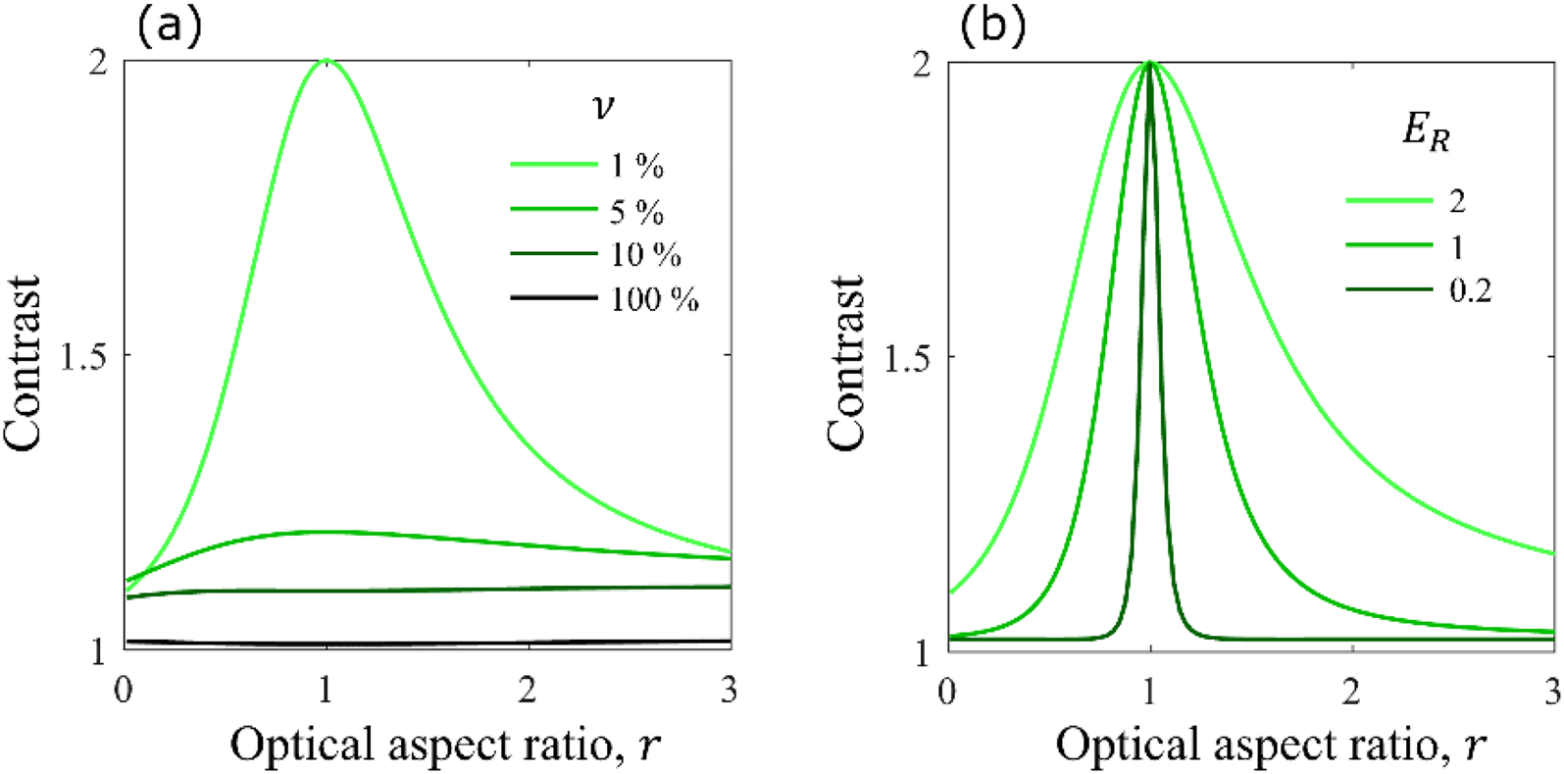
Fluctuating number of particles in the interaction volume. (**a**) $C\left( {r;\nu ,E_{R} = 2} \right)$ and (**b**) $C\left( {r;\nu = 1\% ,E_{R} } \right)$ for a group of 100 particles on average.

In dynamic random media, such as colloids, where particles are diffusively moving and orienting, the number of particles in the interaction volume fluctuates following a Poisson distribution ^14,31^. Unlike the previous case, the scattered intensity now fluctuates due to random orientations, positions and number of particles in the interaction volumes. In supplementary section III, we derive the contrast as

3 $$C\left( {r,\left\langle N \right\rangle ;\nu ,E_{R} } \right) = 1 + \frac{{f\left( {r;\nu ,E_{R} } \right)}}{\left\langle N \right\rangle }$$

for *an average* $\left\langle N \right\rangle$ particles in the total interaction volume. Note that Eq. (3) is similar to Eq. (2) except for the absence of the last term in Eq. (2), which suggests that the contrast for the case with number fluctuations will be greater than that without number fluctuations. The contrast dependence on $r$ is plotted in Fig. 4 for an interaction volume structure similar to the one in Fig. 3.

While the effects of varying $\nu$ and $E_{R}$ on the sensitivity of the contrast to $r$ are similar for constant and fluctuating number of particles, the latter enhances the contrast to values greater than unity. In fact, there is no upper bound for the contrast, which could be an appealing characteristic for practical measurements.

These results suggest that structuring the interaction volume brings the intensity fluctuations into a controllable statistical regime where the information recovery is optimal. In such a “non-gaussian” regime, when the mean and variance of the number of particles in the total interaction volume is known, the shape of randomly positioning and orienting particles can be retrieved by a single measurement of the contrast of intensity fluctuations, which, in principle, only requires the use of a single detector.

## Structured illumination using vector beams

A convenient way to structure the interaction volume is by using so-called “space-polarization classically entangled fields”^32^, i.e., electromagnetic fields who’s field distributions may be described as an inseparable superposition of orthogonal spatial distributions associated with orthogonally polarized states. A specific class of such space-polarization entangled fields are cylindrical vector Bessel beams (CVBs). Any spatial distribution of polarization states, or “state”, of a focused CVB may be decomposed into the basis of radially and azimuthally polarized beams. Upon focusing, only the radially polarized beam acquires a longitudinally polarized spatial distribution while the azimuthally polarized beam has a purely transversal field distribution^33^. In the geometry of Fig. 2, if the analyzer is oriented parallel to the propagation direction, then the longitudinally and transversally polarized regions of the cylindrical vector beam will serve as the co and cross-polarized volumes, respectively.

**Figure 5:**
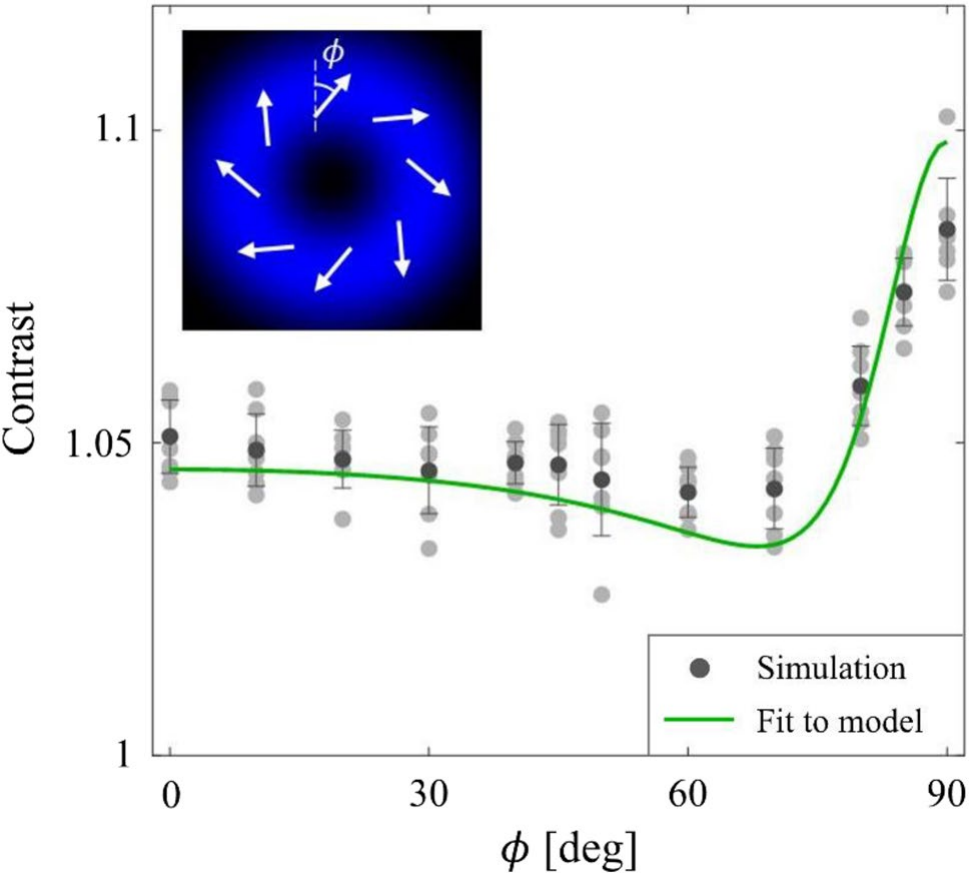
Contrast dependence on CVB illumination. Monte-Carlo simulation of the contrast of intensity fluctuations as a function of incident state of polarization. Also shown with continuous line is the fit to the random walk model (see text). Optical aspect ratio used in the simulation was $r$=1.8 while the fit provides a value of $r$=1.75 $\pm$ 0.29. The inset describes $\phi$, which is the angle between the local polarization state vector and the local radial vector. $\phi$ = 0° corresponds to a radially polarized beam and $\phi$ = 90° corresponds to an azimuthally polarized beam.

To use CVBs within the domain of the proposed model, we first need to find the relationship between the interaction volume structure parameters $\nu$ and $E_{R}$ and the practically tunable parameters of the illumination. For CVBs, these parameters are the angle made by the plane wave components of the beam with the optic axis, ${\Theta }$, and the angle between the local polarization state vector and the local radial vector (a vector pointing radially outward at a given point), $\phi$. As described in the inset of Fig. 5, $\phi$ = 0° corresponds to a radially polarized beam and $\phi$ = 90° corresponds to an azimuthally polarized beam. In supplementary section IV, we show that

4 $$\begin{gathered} \nu \left( {\Theta } \right) \to \nu = 0.5, \hfill \\ E_{R} \left( {{\Theta },\phi } \right) = \tan \left( {\Theta } \right) \cdot \frac{\cos \left( \phi \right)}{{\sqrt {\cos^{2} \left( \phi \right) + \sin^{2} \left( \phi \right)\left( {1 + \tan^{2} \left( {\Theta } \right)} \right)} }} . \hfill \\ \end{gathered}$$

At this point, it is important to note that our theoretical framework considers hard-edged and homogeneously intense interaction volumes. While such an interaction volume enables theoretical analysis, any practical implementation of such an interaction volume will be an approximation. To investigate how well our model describes the contrast of intensity fluctuations from vector beams, we perform a Monte-Carlo simulation for a random particle group with $r$ = 1.80 as described in detail in supplementary section V. Briefly, we illuminate a random group of identical particles by state $\phi$ and calculate the contrast of intensity fluctuations scattered perpendicular to the illumination direction after an analyzer oriented along the propagation direction for several illumination states. By fitting the dependence of contrast on $\phi$ to Eq. (3), we retrieve $r$ and $\left\langle N \right\rangle$. The calculated contrasts and an average of all fits are shown in Fig. 5 as filled circles and the solid line, respectively. After repeating this procedure 7 times, we obtained $r$ = 1.75 $\pm$ 0.29 and $\left\langle N \right\rangle$ = 44 $\pm$ 3 particles. This result suggests that cylindrical vector beams are a viable option to implement the interaction volume considered in our theoretical framework.

## Experimental demonstration

**Figure 6:**
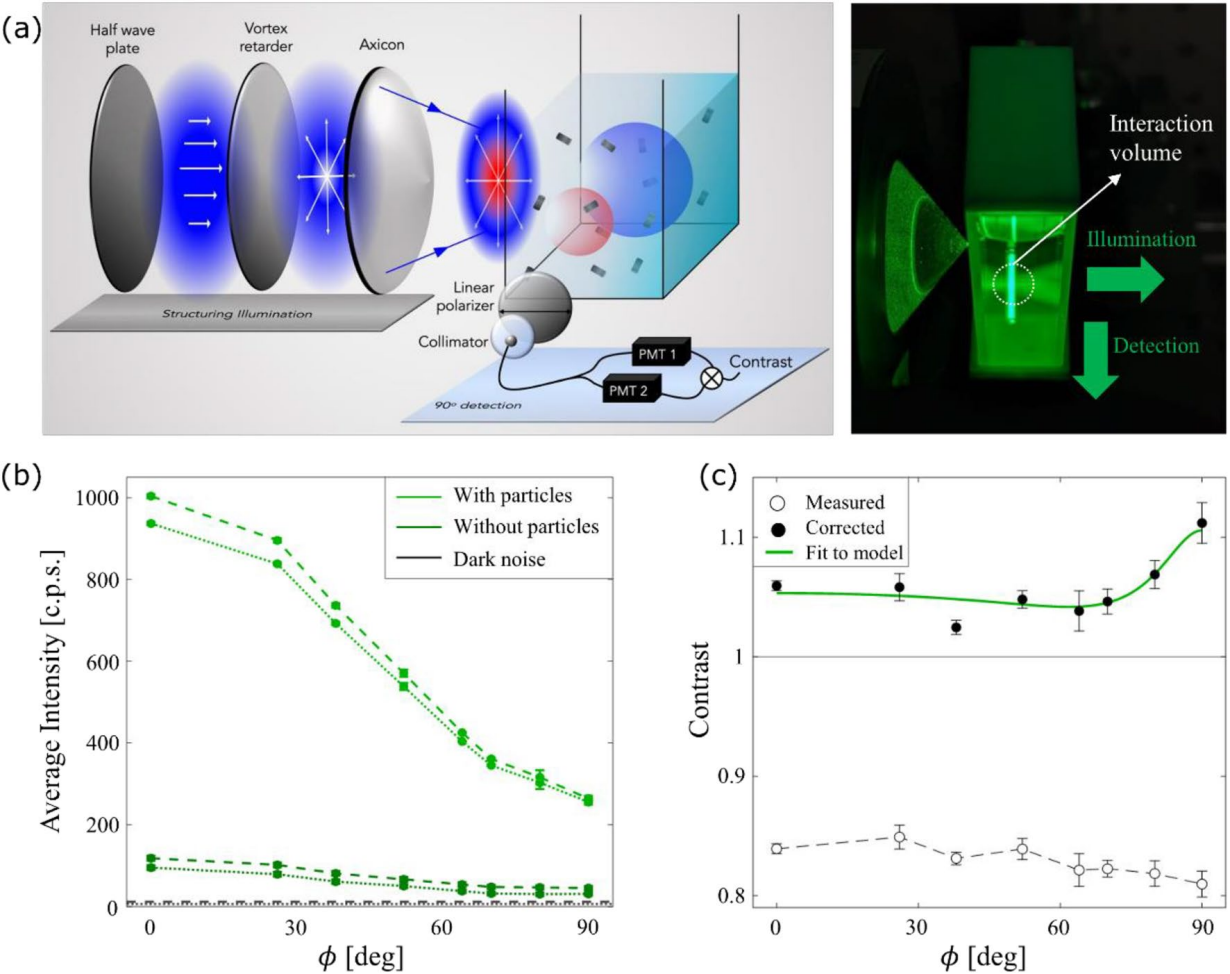
Experimental measurement of nanoparticles shape. (**a**) A focussed CVB state is created using a half-wave plate, vortex retarder and axicon and it illuminates a sparse dispersion (2.5 $\times$ 10^–8^ vol. %) of 30 nm $\times$ 100 nm TiO_2_ nanorods in water. The space-polarization structuring of the illumination creates 2 independent orthogonally polarized interaction volumes. To apply our theoretical framework, we approximate the true interaction volume as two hard edged non-overlapping spheres with adjustable volumes and intensities as depicted by the red and blue spheres. The contrast of intensity fluctuations at a 90° scattering angle and polarized along the direction of illumination are measured for various states of illumination. (**b**) Average intensity as a function of the illumination state in PMT 1 (stroked line) and PMT 2 (dotted line). (**c**) Contrast of intensity fluctuations as a function of the illumination state.

The proposed measurement scheme is practically implemented as schematically described in Fig. 6a. A cylindrical vector Bessel beam with ${\Theta }$ = 30° is created by a combination of a vortex retarder and a large angle axicon. The state of the cylindrical vector beam (radially polarized, azimuthally polarized, or a superposition), which also determines $E_{R}$ as shown in Eq. (4), can be chosen by adjusting the orientation of the vortex retarder. The sample is a sparse dispersion of 30 nm $\times$ 100 nm TiO_2_ nanorods at a volume concentration of 2.5 $\times$ 10^–8^. By approximating the nanorods as spheroidal dipoles, we estimate $r \approx$ 1.83^34^. In supplementary section VII, we show that by approximating the interaction volume as one with 3D gaussian edges^35–37^, $\left\langle N \right\rangle \approx$ 26 particles. To measure the contrast of intensity fluctuations scattered perpendicular to illumination direction for different states of the CVB, we measure the intensity-intensity temporal correlation function, $g^{\left( 2 \right)} \left( \tau \right) = \frac{{\left\langle {I\left( 0 \right)I\left( \tau \right)} \right\rangle }}{{\left\langle {I\left( 0 \right)} \right\rangle \left\langle {I\left( \tau \right)} \right\rangle }} - 1$. Since the contrast is the value of $g^{\left( 2 \right)} \left( \tau \right)$ at $\tau = 0$, we fit the measured correlation function to $a \cdot \exp \left( { - \frac{\tau }{{\tau_{o} }}} \right) + c$ and determine the contrast as $a + c$. A more detailed description of the experimental procedure is given in supplementary section VI.

The measurement procedure was repeated three times and the results are summarized in Fig. 6b and c. Note that while the dynamic range of the measured contrasts is small, the standard deviations of our measurements are even smaller, demonstrating the measurement reliability. As expected, due to the very low intensities, the contrast is diminished to values less than unity, which is typical for measurements with small signal to noise ratios^36,38–41^. In such cases, correcting the contrast for the background noise becomes imperative^40,41^. In our experiment, this noise originates from the unavoidable multiple reflections from the walls of the cuvette and other optical components. In supplementary section VI, we show that if the background noise and the signal are independent random variables, the measured contrast can be corrected to obtain  $C_{true}=\beta^{ - 1} \cdot C_{meas}$ where $\beta^{ - 1} = 1 + \beta_{1}^{ - 1} + \beta_{2}^{ - 1} + \beta_{1}^{ - 1} \beta_{2}^{ - 1}$ and $\beta_{j}$ is the signal to noise ratio in the $j{\text{th}}$ PMT. This correction requires noise measurements for different CVB states from a cuvette filled with clean water, which is shown in Fig. 6b.

By fitting the corrected contrast to Eq. (3), one can retrieve both $r$ and $\left\langle N \right\rangle$. By repeating this procedure three times, we obtain $r =$ 1.93 $\pm$ 0.29, which is within 6% of the estimated value of $r$ based on the manufacturer data. At the same time we infer $\left\langle N \right\rangle =$ 39 $\pm$ 3.5 particles, which is close to the value $\left\langle N \right\rangle \approx$ 26 particles estimated based on considering an isotropic, soft-edged interaction volume. The discrepancy may originate in the approximations used to define the size of this volume as well as possible uncertainties in optical alignment and sample preparation.

To conclude, we note that this methodology is applicable in conditions wherein the polarizability tensor of an individual particle can be safely approximated by a diagonal matrix with at least two equal diagonal elements. Whether the polarizability tensor is diagonal not only depends on the size of the particle, but also on the wavelength of operation and refractive index at that wavelength. In our experiment, we demonstrated that this condition can be satisfied for a particle with a major axis dimension (in our case, 100 nm) approximately a fifth of the operation wavelength (in our case, 532 nm). Secondly, the sensitivity of the contrast to $r$ is inversely proportional to the average *number* of particles in the interaction volume. This parameter depends on both the concentration of the solution and the size of the interaction volume. While, in principle, the sensitivity can be improved by reducing the number of particles in the interaction volume by diluting the sample, focusing the illumination or reducing the angular aperture of the detector, this comes with an unavoidable reduction in the signal to noise ratio. Hence, the experimental setup needs to be implemented to not only increase the number of photons measured by the detectors, but also to reduce the noise which in our setup primarily originates from multiple reflections of light in the cuvette. Lastly, we note that while we choose to use CVBs for experimental convenience, other field structuring techniques could also be used within our theoretical framework. For instance, the interaction volume described in Fig. 2 may be created by splitting a linearly polarized beam into two orthogonally polarized ones with a Wollaston prism and the structure parameters $\nu$ and $E_{R}$ could be modified by varying the sizes of the two beams and the initial polarization state, respectively.

## Discussion and conclusion

Measuring the shape of nanoparticles is a daunting task when one is limited to a group of randomly moving and orienting particles. Furthermore, in many situations, the number of particles in the interaction volume is so large that the measurables start obeying Gaussian statistics and are void of relevant information. Our approach enforces a non-Gaussian regime of interaction where the statistical properties of measurable quantities depend on more parameters than when they are simply Gaussian random variables^28,39^. For example, the contrast of non-Gaussian intensity fluctuations depends on both the shape and number of nanoparticles, whereas that from Gaussian intensity fluctuations depends on neither. Hence, a carefully designed measurement methodology may enable one to retrieve the parameters that determine the statistical moments of the non-Gaussian distribution.

Such a favorable regime of interaction can be established, for instance, by reducing the number of the particles in the interaction volume, by generating a flow in a low concentration colloidal solution^42^, or by measuring fluorescence from a few particles^43^. However, structural modifications of the probed samples may not always be practical, it could be invasive, and most of the time it cannot be actively controlled. In our methodology, instead of modifying the sample, we structure the illumination. Not only does this make our procedure non-invasive, but the field structuring also enables active control of the statistical properties of the scattered intensity, which allows us to retrieve both the optical aspect ratio and number of particles in concentration regimes previously inaccessible by traditional scattering techniques.

Scattering measurements on random media at ultra-low concentrations have statistical outcomes that are often non-Gaussian random variables^28,39^. While here we were concerned with determining the nanoparticle’s shape, we note that our model can serve as a starting point for other problems wherein the final measurable may be modelled as a sum of independent random walks. For instance, the scattering of classically entangled fields^32,44^ from sparse groups of particles could lead to this type of observables. When a particle group has statistically different responses to the different degrees of freedom (polarization state, color, etc.), the overall outcome of the interaction could be modelled as a superposition of independent random walks. A similar measurable quantity could also originate from scattering of unstructured light from a sparse mixture of several different types of particles. At the other extreme, the field resulting from the interference of light trajectories in turbid random media may also deviate from the common Gaussian statistics^40^ and results from this model may be applicable.

Even after establishing a model and measurement methodology to recover the desired information, a critical challenge lies in accurately measuring the contrast of non-Gaussian intensity fluctuations. Nevertheless, we demonstrated that, despite the low scattered intensities that define this regime of interaction, the measured statistical properties can be appropriately corrected for the inherent noise contributions. In a broader context, overcoming this hurdle and providing background free contrast data may allow approaching other kinds of statistical measurements that can exploit non-Gaussian measurables.

### Supplementary Information

Supplementary Information.

## Data Availability

Data underlying the results presented in this paper are not publicly available at this time but may be obtained from the corresponding author upon reasonable request.
