# Supplementary material for: Measuring nanoparticles shape by structured illumination
Source: Sci Rep. 2024 Mar 4;14:5348. doi: 10.1038/s41598-024-53665-1 (PMC10912601; doi:10.1038/s41598-024-53665-1)
Supplement: Supplementary file 1 — Supplementary Information. [file 41598_2024_53665_MOESM1_ESM.docx]

**Supplementary information:**

**Measuring nanoparticles shape by structured illumination**

Shubham Dawda, Zhean Shen, Aristide Dogariu*

[**adogariu@creol.ucf.edu*](mailto:*adogariu@creol.ucf.edu)

*CREOL, The College of Optics and Photonics, 4304 Scorpius Street, Orlando, Florida–32816*

**Table of Contents**

[I. Contrast of intensity fluctuations for 2 fixed randomly orienting anisotropic dipoles under different excitation conditions 2](#_Toc139042168)

[II. Mean and variance of intensity fluctuations from $M$ interaction volumes 5](#_Toc139042169)

[III. Contrast of intensity fluctuations for random group of anisotropic dipoles 8](#_Toc139042170)

[IV. Derivation of $\nu$ and $E_{R}$ for Cylindrical Vector Bessel beams 11](#_Toc139042171)

[V. Monte-Carlo simulation of scattering from CVBs 13](#_Toc139042172)

[VI. Experiment and contrast correction 14](#_Toc139042173)

[VII. Size of interaction volume 16](#_Toc139042174)

[VIII. References 17](#_Toc139042175)

# **I. Contrast of intensity fluctuations for 2 fixed randomly orienting anisotropic dipoles under different excitation conditions**

Consider an anisotropic but axisymmetric dipole with polarizability tensor $\bar{\alpha}=\text{diag}(\alpha_{1},\alpha_{2},\alpha_{2})$. The fields scattered from such a dipole perpendicular to the excitation direction under co and cross polarized excitations are $a_{\parallel}$ and $a_{\times}$, respectively. From^1^, these are

|  | $a_{\parallel}=\left\vert E_{\parallel} \right\vert\alpha_{2}\left( \left( r-1 \right)\sin^{2} \left( \psi\right)\cos^{2} \left( \varphi\right)+1 \right)$  $a_{\times}=\left\vert E_{\times} \right\vert\alpha_{2}\left( r-1 \right)\sin^{2} \left( \psi\right)\sin\left( \varphi\right)\cos\left( \varphi\right)$ | ( S1 ) |
| --- | --- | --- |

where the optical aspect ratio $r=\alpha_{1}/\alpha_{2}$, $\psi$ and $\varphi$ denote the dipole orientation (Figure S1) and $E_{\parallel}$ and $E_{\times}$ are the co and cross polarized excitation field magnitudes, respectively.


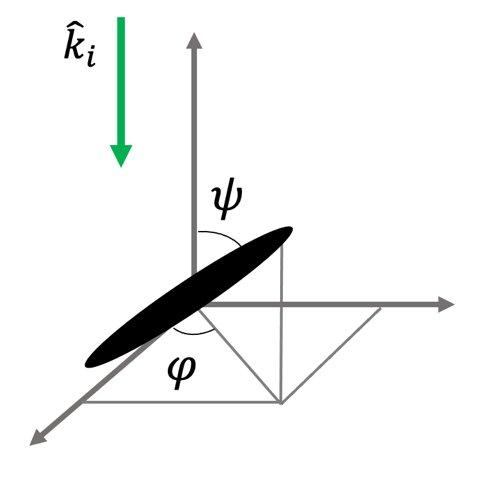


Figure S1: Anisotropic axisymmetric dipole orientations ($\psi,\varphi$) with respect to excitation plane wave direction $\hat{k}_{i}$.

Since the dipoles are randomly orienting, the probability density functions of the orientations would be $p_{\psi}\left( \psi\right)=\frac{\sin\left( \psi\right)}{2}$ and $p_{\varphi}\left( \varphi\right)=\frac{1}{2\pi}$ where $\psi\in\left[ 0,\pi\right]$ and $\varphi\in\left[ 0,2\pi\right]$ and the orientation average can be calculated as

$$\left\langle a\left( \psi,\varphi\right) \right\rangle_{o}=\int_{\psi} \int_{\varphi} a\left( \psi,\varphi\right)p_{\psi}\left( \psi\right)p_{\varphi}\left( \varphi\right)d\varphi d\psi=\frac{1}{4\pi}\int_{0}^{\pi} \left[ \int_{0}^{2\pi} a\left( \psi,\varphi\right)d\varphi\right]\sin\left( \psi\right)d\psi.$$

The first four moments of the scattered fields after such an orientation averaging are

|  | $\left\langle a_{\parallel} \right\rangle_{o}=\left\vert E_{\parallel} \right\vert\alpha_{2}\cdot\left( \frac{2+r}{3} \right)$  $\left\langle a_{\parallel}^{2} \right\rangle_{o}=\left( \left\vert E_{\parallel} \right\vert\alpha_{2} \right)^{2}\cdot\left( \frac{8+4r+3r^{2}}{15} \right)$  $\left\langle a_{\parallel}^{3} \right\rangle_{o}=\left( \left\vert E_{\parallel} \right\vert\alpha_{2} \right)^{3}\cdot\left( \frac{16+8r+6r^{2}+5r^{3}}{35} \right)$  $\left\langle a_{\parallel}^{4} \right\rangle_{o}=\left( \left\vert E_{\parallel} \right\vert\alpha_{2} \right)^{4}\cdot\left( \frac{128+64r+48r^{2}+40r^{3}+35r^{4}}{315} \right)$ | ( S2 ) |
| --- | --- | --- |

and

|  | $\left\langle a_{\times} \right\rangle_{o}=0$  $\left\langle a_{\times}^{2} \right\rangle_{o}=\left( \left\vert E_{\times} \right\vert\alpha_{2} \right)^{2}\cdot\left( \frac{\left( r-1 \right)^{2}}{15} \right)$  $\left\langle a_{\times}^{3} \right\rangle_{o}=0$  $\left\langle a_{\times}^{4} \right\rangle_{o}=\left( \left\vert E_{\times} \right\vert\alpha_{2} \right)^{4}\cdot\left( \frac{\left( r-1 \right)^{4}}{105} \right).$ | ( S3 ) |
| --- | --- | --- |

Next, consider two such anisotropic dipoles that are fixed in space but uniformly randomly and independently orienting. Let the field scattered for the first dipole be $a_{1}$ and that from the second be $a_{2}$. Since the dipoles are fixed in space, there will not be any phase term and the measured intensity will be, $I=\left( a_{1}+a_{2} \right)^{2}$. We want to model $C=\frac{Var\left( I \right)}{\left\langle I \right\rangle^{2}}$ where $\left\langle\ldots\right\rangle$ denotes an average over independent realizations, each realization characterized by uniformly random orientations of both dipoles. Since both dipoles are independently randomly orienting, we can use $\left\langle a_{1}^{l}a_{2}^{m} \right\rangle_{o}=\left\langle a_{1}^{l} \right\rangle_{o}\left\langle a_{2}^{m} \right\rangle_{o}$ ^2^ to derive

|  | $\left\langle I \right\rangle=\left\langle a_{1}^{2} \right\rangle_{o}+\left\langle a_{2}^{2} \right\rangle_{o}+2\left\langle a_{1} \right\rangle_{o}\left\langle a_{2} \right\rangle_{o}$  $\left\langle I^{2} \right\rangle=\left\langle a_{1}^{4} \right\rangle_{o}+\left\langle a_{2}^{4} \right\rangle_{o}+6\left\langle a_{1}^{2} \right\rangle_{o}\left\langle a_{2}^{2} \right\rangle_{o}+4\left\langle a_{1}^{3} \right\rangle_{o}\left\langle a_{2} \right\rangle_{o}+4\left\langle a_{1} \right\rangle_{o}\left\langle a_{2}^{3} \right\rangle_{o}.$ | ( S4 ) |
| --- | --- | --- |

Case I: Both excitations are polarized parallel to the analyzer ($\left\langle a_{1}^{m} \right\rangle=\left\langle a_{2}^{m} \right\rangle=\left\langle a_{\parallel}^{m} \right\rangle$)

$$Contrast=\frac{Var\left( I \right)}{\left\langle I \right\rangle^{2}}(a_{1}=a_{2}=a_{\parallel})=\frac{4\left( r-1 \right)^{2}\left( 101r^{2}+278r+251 \right)}{7\left( 22+16+7r^{2} \right)^{2}}$$

Case II: Both excitations are polarized orthogonal to the analyzer ($\left\langle a_{1}^{m} \right\rangle=\left\langle a_{2}^{m} \right\rangle=\left\langle a_{\times}^{m} \right\rangle$)

$$Contrast=\frac{Var\left( I \right)}{\left\langle I \right\rangle^{2}}(a_{1}=a_{2}=a_{\times})=\frac{11}{7}$$

Case III: One excitations is polarized parallel and the other is polarized orthogonal to the analyzer ($\left\langle a_{1}^{m} \right\rangle=\left\langle a_{\parallel}^{m} \right\rangle$, $\left\langle a_{2}^{m} \right\rangle=\left\langle a_{\times}^{m} \right\rangle$)

$$Contrast=\frac{Var\left( I \right)}{\left\langle I \right\rangle^{2}}\left( a_{1}=a_{\parallel},a_{2}=a_{\times} \right)=\frac{4\left( r-1 \right)^{2}\left\{ 2\left( r-1 \right)^{2}+7E_{R}^{2}\left( 8+4r+3r^{2} \right)+4E_{R}^{4}\left( 12+16r+7r^{2} \right) \right\}}{7\left( \left( r-1 \right)^{2}+E_{R}^{2}\left( 8+4r+3r^{2} \right) \right)^{2}}$$

where $E_{R}=\frac{\left| E_{\parallel} \right|}{\left| E_{\times} \right|}$.

To verify the equations derived above, we perform Monte-Carlo simulations. We first generate 50,000 independent orientations of both dipoles with various $r$s and calculate the scattered fields from each under co and cross-polarized excitations using ( S1 ). We calculate the measured intensity as the square magnitude of the sum of both scattered fields. This results in 50,000 values of intensity for each $r$ in each of the 3 cases. We calculate the contrast over the 50,000 values of intensity. The simulated and modelled contrasts for all three cases are plotted in Figure S2 where agreement is noted.


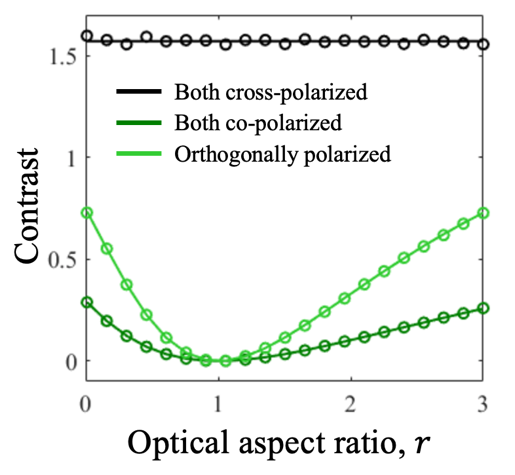


Figure S2: Comparison of modelled (solid line) and simulated (empty dots) Contrast($r$).

# **II. Mean and variance of intensity fluctuations from** $M$ **interaction volumes**

The scattered field from the total interaction volume is the coherent superposition of scattered fields from each of the $M$ sub-interaction volumes–

$$A\exp\left( i\Phi\right)=\sum_{m=1}^{M} A_{m}exp(i\Phi_{m}).$$

For simplicity, we’ll first consider the specific case of 3 interaction volumes and then generalize the final results to $M$ interaction volumes. The final intensity, $I$, is given as

|  | $I=A^{2}=\sum_{m=1}^{3} A_{m}^{2}+2A_{1}A_{2}\cos\left( \Phi_{1}-\Phi_{2} \right)+2A_{2}A_{3}\cos\left( \Phi_{2}-\Phi_{3} \right)+2A_{1}A_{3}\cos\left( \Phi_{1}-\Phi_{3} \right).$ | ( S5 ) |
| --- | --- | --- |

Using the following three assumptions, we’ll analyze ( S5 ) and derive the general equation for the mean and variance of the intensity scattered from the total interaction volume:

|  | $\Phi_{k}\sim Uniform\left( 0,2\pi\right)\text{ for all }k$ | ( A1 ) |
| --- | --- | --- |
|  | $\left\langle A_{j}A_{k} \right\rangle=\left\langle A_{j} \right\rangle\left\langle A_{k} \right\rangle$ and $\left\langle\Phi_{j}\Phi_{k} \right\rangle=\left\langle\Phi_{j} \right\rangle\left\langle\Phi_{k} \right\rangle$ for $j\neq k$ | ( A2 ) |
|  | $\left\langle A_{j}\Phi_{k} \right\rangle=\left\langle A_{j} \right\rangle\left\langle\Phi_{k} \right\rangle$ for all $j$ and $k$ | ( A3 ) |

where $\left\langle\ldots\right\rangle$ denotes an average over independent realizations.

**Modelling** $\left\langle I \right\rangle$**:**

From ( A1 ) and ( A3 ), the last 3 terms in ( S5 ) vanish to give

$$\left\langle I \right\rangle=\sum_{m=1}^{3} \left\langle A_{m}^{2} \right\rangle=\sum_{m=1}^{3} \left\langle I_{m} \right\rangle.$$

Hence, for $M$ interaction volumes

|  | $\left\langle I \right\rangle=\sum_{m=1}^{M} \left\langle I_{m} \right\rangle.$ |  |
| --- | --- | --- |

**Modelling** $Var(I)$**:**

It is a well-known statistical property that if $Z_{j}$ are independent random variables, then

|  | $Var\left( \sum_{j=1}^{J} Z_{j} \right)=\sum_{j=1}^{J} Var\left( Z_{j} \right)$ | ( S6 ) |  |
| --- | --- | --- | --- |

In order to apply this property in our model, we need to check independence of the terms in ( S5 ). To do so, we’ll use the fact that if random variables $A$ and $B$ are independent, then $\left\langle AB \right\rangle=\left\langle A \right\rangle\left\langle B \right\rangle$ and in the following show that all terms in ( S5 ) are statistically independent from each other.

**Checking independence among the first 3 terms in** ( S5 )**:**

Since interaction volumes are non-overlapping, scattered fields from each will be independent of each other:

$$\left\langle A_{1}^{2}A_{2}^{2} \right\rangle=\left\langle A_{1}^{2} \right\rangle\left\langle A_{2}^{2} \right\rangle$$

Similar result can be shown for all other pairs. Hence, product of first 3 terms are independent.

**Checking independence among the first 3 and last 3 terms in** ( S5 )**:**

Since $A$s and $\Phi$s are independent and $\Phi$s are uniformly distributed,

$$\left\langle A_{1}^{2}\cdot2A_{1}A_{2}\cos\left( \Phi_{1}-\Phi_{2} \right) \right\rangle=\left\langle A_{1}^{2}\cdot2A_{1}A_{2} \right\rangle\left\langle\cos\left( \Phi_{1}-\Phi_{2} \right) \right\rangle=0$$

$$\left\langle A_{1}^{2} \right\rangle\left\langle2A_{1}A_{2}\cos\left( \Phi_{1}-\Phi_{2} \right) \right\rangle=0$$

Hence,

$$\left\langle A_{1}^{2}\cdot2A_{1}A_{2}\cos\left( \Phi_{1}-\Phi_{2} \right) \right\rangle=\left\langle A_{1}^{2} \right\rangle\left\langle2A_{1}A_{2}\cos\left( \Phi_{1}-\Phi_{2} \right) \right\rangle$$

Similar results can be shown for all other pairs. Hence, first and last terms are independent.

**Checking independence among the last 3 terms in** ( S5 )**:**

$$\left\langle2A_{1}A_{2}\cos\left( \Phi_{1}-\Phi_{2} \right)\cdot2A_{2}A_{3}\cos\left( \Phi_{2}-\Phi_{3} \right) \right\rangle=\left\langle4A_{1}A_{3}A_{2}^{2} \right\rangle\langle\cos\left( \Phi_{1}-\Phi_{2} \right)\cos\left( \Phi_{2}-\Phi_{3} \right)\rangle$$

Noting that:

$$\left\langle\cos\left( \Phi_{1}-\Phi_{2} \right)\cdot\cos\left( \Phi_{2}-\Phi_{3} \right) \right\rangle=0$$

Similar results can be shown for all other pairs. Hence, products of last 3 terms are also independent.

Hence, ( S6 ) can be used to model the variance as

$$Var\left( I \right)=\sum_{m=1}^{3} Var\left( A_{m}^{2} \right)+Var\left( 2A_{1}A_{2}\cos\left( \Phi_{1}-\Phi_{2} \right) \right)+Var\left( 2A_{2}A_{3}\cos\left( \Phi_{2}-\Phi_{3} \right) \right)+Var\left( 2A_{1}A_{3}\cos\left( \Phi_{1}-\Phi_{3} \right) \right)$$

From the definition of variance,

$$Var\left( 2A_{1}A_{2}\cos\left( \Phi_{1}-\Phi_{2} \right) \right)=\left\langle4A_{1}^{2}A_{2}^{2}\cos^{2} \left( \Phi_{1}-\Phi_{2} \right) \right\rangle-\left\langle2A_{1}A_{2}\cos\left( \Phi_{1}-\Phi_{2} \right) \right\rangle^{2}.$$

From ( A1 ) and ( A3 ), the second term vanishes. Since scattered field magnitudes and phases are independent, the first term can be written as $\left\langle4A_{1}^{2}A_{2}^{2} \right\rangle\left\langle\cos^{2} \left( \Phi_{1}-\Phi_{2} \right) \right\rangle$. From ( A1 ), $\left\langle\cos^{2} \left( \Phi_{1}-\Phi_{2} \right) \right\rangle=\frac{1}{2}$ making $Var\left( 2A_{1}A_{2}\cos\left( \Phi_{1}-\Phi_{2} \right) \right)=2\left\langle A_{1}^{2} \right\rangle\left\langle A_{2}^{2} \right\rangle$.

Hence, for 3 interaction volumes, the variance is

$$Var\left( I \right)=\sum_{m=1}^{3} Var\left( A_{m}^{2} \right)+2\left\langle A_{1}^{2} \right\rangle\left\langle A_{2}^{2} \right\rangle+2\left\langle A_{2}^{2} \right\rangle\left\langle A_{3}^{2} \right\rangle+2\left\langle A_{1}^{2} \right\rangle\left\langle A_{3}^{2} \right\rangle.$$

Denoting $I_{m}=A_{m}^{2}$ and by rearranging the terms, the above equation can be written as

$$Var\left( I \right)=\left\langle I \right\rangle^{2}+\sum_{m=1}^{M} \left\{ \left\langle I_{m}^{2} \right\rangle-2\left\langle I_{m} \right\rangle^{2} \right\}.$$

# **III. Contrast of intensity fluctuations for random group of anisotropic dipoles**

Our purpose is to find the relationship between moments of the scattered intensity ($I$) from the total interaction volume and those of the single particle scattered fields ($a$) in the sub-volumes. Since the interaction volumes are independent, the moments of the total scattered intensity ($I$) are related to those of scattered intensities from each sub-volume ($I_{m}$) by equations derived in section II of this supplement. Since the moments of $I_{m}$ are related to those of $a$, once we find this relationship, we can accomplish our primary purpose. In this section, we’ll first use results from random-walk theory to relate the moments of $I_{m}$ to those of $a_{n,m}$ and then derive explicit equations relating $I$ and $a_{n,m}$.

$I_{m}$ is the square of the magnitude of the resultant of a phasor sum

$$I_{m}=\left| \sum_{n=1}^{N_{m}} a_{n,m}\exp\left( i\phi_{n,m} \right) \right|^{2}.$$

When the assumptions ( A1 ) - ( A3 ) are applicable to $a_{m}$ and $\phi_{m}$, the following relations hold^2^,

|  | $\left\langle I_{m} \right\rangle=\left\langle N_{m} \right\rangle\left\langle a_{m}^{2} \right\rangle_{o}$  $\left\langle I_{m}^{2} \right\rangle=\left\langle N_{m} \right\rangle\left\langle a_{m}^{4} \right\rangle_{o}+2\left\langle N_{m}\left( N_{m}-1 \right) \right\rangle\left\langle a_{m}^{2} \right\rangle_{o}^{2}$ | ( S7 ) |  |
| --- | --- | --- | --- |

where $\left\langle\ldots\right\rangle$ represents an average over independent realizations. From equations derived in section II of the supplement, for the specific case of $M=2$ independent interaction volumes, the contrast is

$$C=\frac{Var\left( I \right)}{\left\langle I \right\rangle^{2}}=1+\frac{\sum_{m=1}^{2} \left\{ \left\langle I_{m}^{2} \right\rangle-2\left\langle I_{m} \right\rangle^{2} \right\}}{\left( \sum_{m=1}^{2} \left\langle I_{m} \right\rangle\right)^{2}}.$$

Using equation ( S7 ),

|  | $C=1+\frac{\sum_{m=1}^{2} \left\{ \left\langle N_{m} \right\rangle\left\langle a_{m}^{4} \right\rangle_{o}+2\left\langle a_{m}^{2} \right\rangle_{o}^{2} (\left\langle N_{m}^{2} \right\rangle-\left\langle N_{m} \right\rangle-\left\langle N_{m} \right\rangle^{2}) \right\}}{\left( \sum_{m=1}^{2} \left\langle N_{m} \right\rangle\left\langle a_{m}^{2} \right\rangle_{o} \right)^{2}}.$ | ( S8 ) |  |
| --- | --- | --- | --- |

Note that–

1. Until now, we have not imposed a physical meaning on $a$ as derived in equation ( S8 ). This means that ( S8 ) may be used to model problems other than the one being discussed in this work, and hence is general in that sense.
2. When we consider $a$ to be the scattered field from a single particle, we have not imposed any restriction on the probability distributions of the particle orientations and number of particles in the $m^{th}$ interaction volume. The distributions of $a$ and $N$ could be different in different interaction volumes. The only assumption is that all particles in the $m^{th}$ interaction volume must have the same orientation and number distributions.

For anisotropic dipolar particles that are uniformly randomly orienting, we can use moments derived in ( S2 ) and ( S3 ) for the moments of $a$. Since the two interaction volumes under consideration are the co and cross polarized volumes, we make the change of notation $a_{1},N_{1}\to a_{\parallel},N_{\parallel}$ and $a_{2},N_{2}\to a_{\times},N_{\times}$. The total interaction volume has $N$ particles and because of the interaction volume structuring they’re divided as $N_{\parallel}=\nu N$ and $N_{\times}=\left( 1-\nu\right)N$. Consider 2 specific distributions of the number of particles–

**Case 1: Number of particles is constant (** $\boldsymbol{p}_{\boldsymbol{N}}\left( \boldsymbol{N} \right)\boldsymbol{=\delta}\left( \boldsymbol{N-}\boldsymbol{N}_{\boldsymbol{0}} \right)$ **)**

In this case, all averages around the number in ( S8 ) disappear. This makes the contrast

$$C\left( r,N_{0};\nu,E_{R} \right)=1+\frac{f\left( r;\nu,E_{R} \right)}{N_{0}}-\frac{g\left( r;\nu,E_{r} \right)}{N_{0}}$$

where

|  | $f\left( r;\nu,E_{R} \right)=\frac{\nu\left\langle a_{\parallel}^{4} \right\rangle_{o}+\left( 1-\nu\right\rangle\left\langle a_{\times}^{4} \right\rangle_{o}}{\left( \nu\left\langle a_{\parallel}^{2} \right\rangle_{o}+\left( 1-\nu\right)\left\langle a_{\times}^{2} \right\rangle_{o} \right)^{2}}$  $=\frac{5}{7}\cdot\frac{E_{R}^{4}\nu\left( 128+64r+48r^{2}+40r^{3}+35r^{4} \right)+3\left( 1-\nu\right)\left( r-1 \right)^{4}}{\left( E_{R}^{2}\nu\left( 8+4r+3r^{2} \right)+\left( 1-\nu\right)\left( r-1 \right)^{2} \right)^{2}}$  $g\left( r;\nu,E_{r} \right)=\frac{2\left( \nu\left\langle a_{\parallel}^{2} \right\rangle_{o}^{2}+\left( 1-\nu\right)\left\langle a_{\times}^{2} \right\rangle_{o}^{2} \right)}{\left( \nu\left\langle a_{\parallel}^{2} \right\rangle_{o}+\left( 1-\nu\right)\left\langle a_{\times}^{2} \right\rangle_{o} \right)^{2}}=2\cdot\frac{E_{R}^{4}\nu\left( 8+4r+3r^{2} \right)^{2}+\left( 1-\nu\right)\left( r-1 \right)^{4}}{\left( E_{R}^{2}\nu\left( 8+4r+3r^{2} \right)+\left( 1-\nu\right)\left( r-1 \right)^{2} \right)^{2}}.$ | ( S9 ) |  |
| --- | --- | --- | --- |

To verify ( S9 ), we perform Monte-Carlo simulations as follows. We generate 10,000 realizations of 20 random oriented and distributed particles. Due to the field structuring, $\nu\cdot20$ particles are under co-polarized and $\left( 1-\nu\right)\cdot20$ are under cross-polarized illuminations with the ratio of illumination field magnitudes $E_{R}=\frac{\left| E_{\parallel} \right|}{\left| E_{\times} \right|}=2$. The final intensity is calculated as the magnitude square of the fields scattered by these particles (calculated using ( S1 ) ). From this set of random intensities, we calculate the contrast and compare it to that expected from the model in Figure S3. Good agreement is observed.


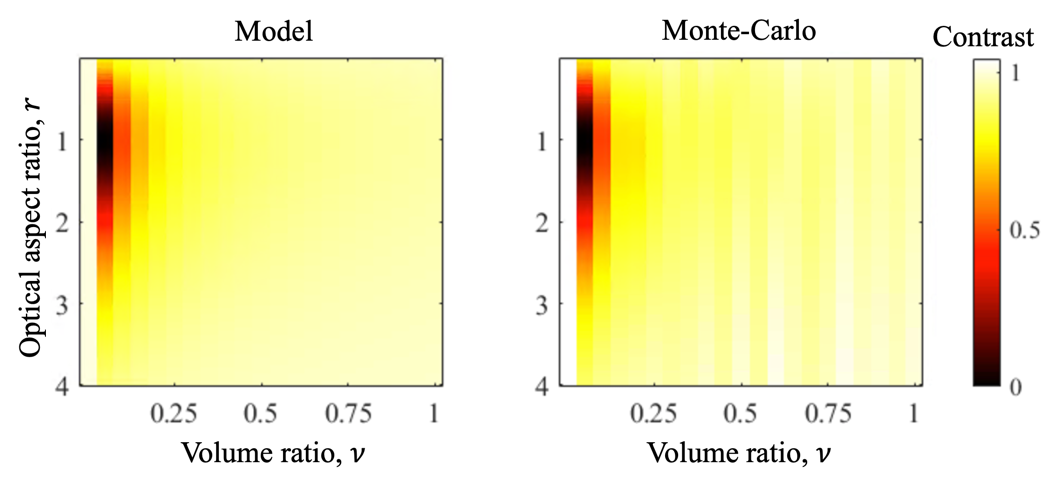


Figure S3: Comparison of modelled and simulated Contrast($r;\nu,E_{R}=2$) for constantly 20 particles in the interaction volume.

**Case 2: Number of particles is Poisson distributed with parameter** $\left\langle\boldsymbol{N} \right\rangle$**: (** $\boldsymbol{p}_{\boldsymbol{N}}\left( \boldsymbol{N} \right)\boldsymbol{=}\frac{\left\langle\boldsymbol{N} \right\rangle^{\boldsymbol{N}}\exp\left( \boldsymbol{-}\left\langle\boldsymbol{N} \right\rangle\right)}{\boldsymbol{N!}}$ **)**

If the number of particles in the total interaction volume follows Poisson statistics, then so should the number of particles in each sub-volume. Since the mean and variance of a Poisson distributed random variable are equal, $\left\langle N_{m}^{2} \right\rangle-\left\langle N_{m} \right\rangle-\left\langle N_{m} \right\rangle^{2}=Var\left( N_{m} \right)-\left\langle N_{m} \right\rangle=0$ and the contrast is

$$C\left( r,\left\langle N \right\rangle;\nu,E_{R} \right)=1+\frac{f\left( r;\nu,E_{R} \right)}{\left\langle N \right\rangle}$$

where $f\left( r;\nu,E_{R} \right)$ is as derived in ( S9 ). To verify this equation, we perform Monte-Carlo simulations as follows. We generate 200,000 realizations of 20 random oriented and distributed particles. Due to the field structuring, $\nu\cdot20$ particles are under co-polarized *on average* and $\left( 1-\nu\right)\cdot20$ are under cross-polarized illuminations *on average* with the ratio of illumination field magnitudes $E_{R}=\frac{\left| E_{\parallel} \right|}{\left| E_{\times} \right|}=0.1$. The final intensity is calculated as the magnitude square of the fields scattered by these particles (calculated using ( S1 ) ). From this set of random intensities, we calculate the contrast and compare it to that expected from the model in Figure S4. Good agreement is observed.


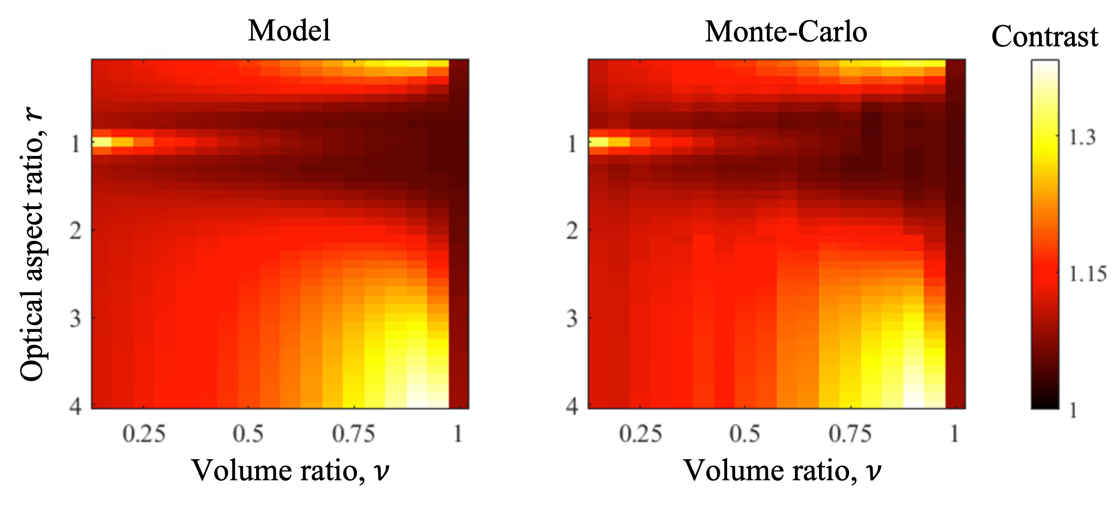


Figure S4: Comparison of modelled and simulated Contrast($r;\nu,E_{R}=0.1$) for on average 20 particles in the interaction volume.

# **IV. Derivation of** $\nu$ **and** $E_{R}$ **for Cylindrical Vector Bessel beams**

The field distribution of a generic cylindrical vector beam has a transversally ($\hat{t}$) and longitudinally ($\hat{z}$) polarized component which can be represented in a cylindrical co-ordinate system ($r,\theta,z$) as $\vec{E}_{CVB}\propto J_{0}\left( k_{t}r \right)\hat{t}+J_{1}\left( k_{t}r \right)\hat{z}$ where $k_{t}=k_{0}\sin\left( \Theta\right)$^3^ and $\Theta$ is the angle made by the propagation direction of the plane wave components of the Bessel beam with the optic axis. Since $\nu$ is the ratio of the longitudinally polarized interaction volume to the total interaction volume,

$$\nu\left( \Theta\right)=\frac{\int_{0}^{\infty} J_{0}\left( k_{t}r \right)^{2}rdr\cdot\int_{-\infty}^{\infty} \exp\left( -\frac{z^{2}}{w_{z}^{2}} \right)dz}{\left( \int_{0}^{\infty} J_{0}\left( k_{t}r \right)^{2}rdr+\int_{0}^{\infty} J_{1}\left( k_{t}r \right)^{2}rdr \right)\int_{-\infty}^{\infty} \exp\left( -\frac{z^{2}}{w_{z}^{2}} \right)dz}$$

$$\nu=\frac{\int_{0}^{\infty} J_{0}\left( k_{t}r \right)^{2}rdr}{\int_{0}^{\infty} J_{0}\left( k_{t}r \right)^{2}rdr+\int_{0}^{\infty} J_{1}\left( k_{t}r \right)^{2}rdr}.$$

Note that since the longitudinal extent of the interaction volume is equal for both the co and cross-polarized volumes, it cancels out and $\nu$ only depends on the transversal field distributions. To solve the above equation, we multiply the field distribution by an apodizing gaussian function and take the limit where the width of the gaussian $\to\infty$.

$$\nu\left( \Theta\right)=\lim_{r_{0}\to\infty} \frac{\int_{0}^{\infty} \exp\left( -\frac{r^{2}}{r_{0}^{2}} \right)J_{0}^{2}\left( k_{t}r \right)rdr}{\int_{0}^{\infty} \exp\left( -\frac{r^{2}}{r_{0}^{2}} \right)J_{0}^{2}\left( k_{t}r \right)rdr+\int_{0}^{\infty} \exp\left( -\frac{r^{2}}{r_{0}^{2}} \right)J_{1}^{2}\left( k_{t}r \right)rdr}.$$

Using $\int_{0}^{\infty} J_{\chi}\left( k_{t}r \right)^{2}\exp\left( -\frac{r^{2}}{r_{0}^{2}} \right)rdr=\frac{r_{0}^{2}}{2}\exp\left( -\frac{1}{2}r_{0}^{2}k_{t}^{2} \right)I_{\chi}\left( \frac{k_{t}^{2}r_{0}^{2}}{2} \right)$ ^4^, we can rewrite the above equation as

$$\nu\left( \Theta\right)=\lim_{\eta\to\infty} \frac{I_{0}(\eta^{2})}{I_{0}(\eta^{2})+I_{1}\left( \eta^{2} \right)}$$

where $\eta^{2}=\frac{k_{t}^{2}r_{0}^{2}}{2}$. Since $\eta$ is large, we can approximate $I_{\chi}\left( \eta^{2} \right)\approx\frac{\exp\left( \eta^{2} \right)}{\sqrt{2\pi\eta^{2}}}\left\{ 1+O\left( \frac{1}{\eta^{2}} \right) \right\}$ ^5^ where $\lim_{\eta\to\infty}O\left( \frac{1}{\eta^{2}} \right)=0$, to re-write the above equation as

$$\nu\left( \Theta\right)=\lim_{\eta\to\infty} \frac{\frac{e^{\eta^{2}}}{\sqrt{2\pi\eta^{2}}}\left\{ 1+O\left( \frac{1}{\eta^{2}} \right) \right\}}{\frac{e^{\eta^{2}}}{\sqrt{2\pi\eta^{2}}}\left\{ 1+O\left( \frac{1}{\eta^{2}} \right) \right\}+\frac{e^{\eta^{2}}}{\sqrt{2\pi\eta^{2}}}\left\{ 1+O\left( \frac{1}{\eta^{2}} \right) \right\}}\approx0.5 .$$

To estimate $E_{R}$, we start from equations of radially and azimuthally polarized Bessel beams as derived in^3^

$$\vec{E}_{RAD}\left( r,\theta,z;\Theta\right)=e^{ik_{z}z}\left[ \begin{matrix} k_{z}\cos\left( \theta\right)J_{1}\left( k_{t}r \right) \\ k_{z}\sin\left( \theta\right)J_{1}\left( k_{t}r \right) \\ k_{t}J_{0}\left( k_{t}r \right) \end{matrix} \right]\cdot\left[ \begin{matrix} \hat{x} \\ \hat{y} \\ \hat{z} \end{matrix} \right],$$

$$\vec{E}_{AZ}\left( r,\theta,z;\Theta\right)=e^{ik_{z}z}\cdot c_{0}\left( \Theta\right)\left[ \begin{matrix} k_{z}\sin\left( \theta\right)J_{1}\left( k_{t}r \right) \\ k_{z}\cos\left( \theta\right)J_{1}\left( k_{t}r \right) \\ 0 \end{matrix} \right]\cdot\left[ \begin{matrix} \hat{x} \\ \hat{y} \\ \hat{z} \end{matrix} \right]$$

where $k_{t}=k_{0}\sin\left( \Theta\right)$, $k_{z}=k_{0}\cos\left( \Theta\right)$ and $c_{0}\left( \Theta\right)$ is a normalization constant such that $\int_{0}^{2\pi} \int_{0}^{\infty} \left| \vec{E}_{RAD} \right|^{2}\left( r,\theta;\Theta\right)rdrd\theta=\int_{0}^{2\pi} \int_{0}^{\infty} \left| \vec{E}_{AZ} \right|^{2}\left( r,\theta;\Theta\right)rdrd\theta$ making $c_{0}^{2}\left( \Theta\right)=1+\frac{k_{t}^{2}}{k_{z}^{2}}=1+\tan^{2} \left( \Theta\right)$. Since the variable $z$ only appears in the phase, the existence of $e^{ik_{z}z}$ is assumed and dropped in the following for brevity. Any cylindrical vector beam state can be represented as a superposition of radially and azimuthally polarized beams as

$$\vec{E}_{CVB}\left( r,\theta;\phi,\Theta\right)=\sin\left( \phi\right)\vec{E}_{AZ}\left( r,\theta;\Theta\right)+\cos\left( \phi\right)\vec{E}_{RAD}\left( r,\theta;\Theta\right)$$

|  | $\vec{E}_{CVB}\left( r,\theta;\phi,\Theta\right)=\left[ \begin{matrix} E_{x} \\ E_{y} \\ E_{z} \end{matrix} \right]\cdot\left[ \begin{matrix} \hat{x} \\ \hat{y} \\ \hat{z} \end{matrix} \right]$  $=\left[ \begin{matrix} \left( \cos\left( \phi\right)\cos\left( \theta\right)+c_{0}\left( \Theta\right)\sin\left( \phi\right)\sin\left( \theta\right) \right)k_{z}J_{1}\left( k_{t}r \right) \\ \left( \sin\left( \phi\right)\cos\left( \theta\right)+c_{0}\left( \Theta\right)\sin\left( \phi\right)\cos\left( \theta\right) \right)k_{z}J_{1}\left( k_{t}r \right) \\ \cos\left( \phi\right)k_{t}J_{0}\left( k_{t}r \right) \end{matrix} \right]\cdot\left[ \begin{matrix} \hat{x} \\ \hat{y} \\ \hat{z} \end{matrix} \right].$ | ( S10 ) |  |
| --- | --- | --- | --- |

Since $E_{R}\left( r,\theta;\phi,\Theta\right)$ is the ratio of the co-polarized field magnitude to the cross-polarized field magnitude, it can be calculated as

$$E_{R}^{2}\left( r,\theta;\phi,\Theta\right)=\frac{\int_{0}^{2\pi} \int_{0}^{\infty} \left| E_{z} \right|^{2}\left( r,\theta;\phi,\Theta\right) rdrd\theta}{\int_{0}^{2\pi} \int_{0}^{\infty} \left\{ \left| E_{x} \right|^{2}\left( r,\theta;\phi,\Theta\right)+\left| E_{y} \right|^{2}\left( r,\theta;\phi,\Theta\right) \right\}rdrd\theta}.$$

From ( S10 ), $\int_{0}^{2\pi} \int_{0}^{\infty} \left| E_{z} \right|^{2} rdrd\theta=2\pi k_{t}\cos^{2} \left( \phi\right)\int_{0}^{\infty} J_{0}^{2}\left( k_{t}r \right)rdr$ and $\int_{0}^{2\pi} \int_{0}^{\infty} \left\{ \left| E_{x} \right|^{2}+\left| E_{y} \right|^{2} \right\}rdrd\theta=2\pi k_{z}^{2}\left[ \cos^{2} \left( \phi\right)+c_{0}^{2}\sin^{2} \left( \phi\right) \right]\int_{0}^{\infty} J_{1}^{2}\left( ar \right)rdr$. Hence,

$$E_{R}^{2}\left( r,\theta;\phi,\Theta\right)=\frac{k_{t}^{2}}{k_{z}^{2}}\cdot\frac{\cos^{2} \left( \phi\right)}{\cos^{2} \left( \phi\right)+c_{0}^{2}\sin^{2} \left( \phi\right)}\cdot\frac{\int_{0}^{\infty} J_{0}^{2}\left( ar \right)rdr}{\int_{0}^{\infty} J_{1}^{2}\left( ar \right)rdr}.$$

As shown in the derivation of $\nu$, $\frac{\int_{0}^{\infty} J_{0}^{2}\left( ar \right)rdr}{\int_{0}^{\infty} J_{1}^{2}\left( ar \right)rdr}=1$, giving

$$E_{R}\left( r,\theta;\phi,\Theta\right)=\tan\left( \Theta\right)\frac{\cos\left( \phi\right)}{\sqrt{\cos^{2} \left( \phi\right)+\left( 1+\tan^{2} \left( \Theta\right) \right)\sin^{2} \left( \phi\right)}} .$$

# **V. Monte-Carlo simulation of scattering from CVBs**

Axisymmetric anisotropic dipoles with $r=1.8$ and random orientations ($\psi,\varphi$) and positions are generated within a virtual box of $40 \mu$m in the $\hat{x}$ and $\hat{y}$ directions and 3500 $\mu$m in the $\hat{z}$ direction. The number of dipoles in the box, $N$, fluctuates following a Poisson distribution^6^ with an average of 107 particles. The dipole group is excited by a CVB of state $\phi$ propagating along $\hat{z}$.

The vectorial excitation field at the position of the $n^{th}$ dipole is calculated using ( S10 ). In response to each vectorial component of this excitation, the $n^{th}$ dipole scatters a field at 90 degrees polarized along $ẑ$. Hence, the field scattered from the $n^{th}$ dipole, $a_{n}e^{i\phi_{n}}$ is calculated as $a_{n}e^{i\phi_{n}}=\left( a_{x\to z}+a_{y\to z}+a_{z\to z} \right)e^{i\phi_{n}}$ where $a_{z\to z}$ is calculated using $a_{\parallel}$ and $a_{x\to z}$ and $a_{y\to z}$ are calculated using $a_{\times}$ as derived in ( S1 ). $\phi_{n}$ is the phase acquired by the scattered field in propagating from the dipole to the detector (placed 1 m away). This is done for all dipoles in the group and the intensity scattered by the group is calculated as $\left| \sum_{n=1}^{N} a_{n}\exp\left( i\phi_{n} \right) \right|^{2}$. This procedure is repeated for 100,000 independent random dipole groups resulting in 100,000 values of scattered intensities for each illumination state ($\phi$). The contrast of intensity fluctuations is calculated as $Contrast\left( \phi\right)=\frac{Var\left( I\left( \phi\right) \right)}{\left\langle I\left( \phi\right) \right\rangle^{2}}$ where $I\left( \phi\right)$ is the set of 100,000 intensities calculated for state $\phi$. The above procedure is repeated 7 times resulting in 7 values of the contrast for each $\phi$ which are plotted as light filled circles in Figure 5 of the main text. The average and standard deviation calculated from these 7 values of contrast are plotted as dark filled circles with error bars in Figure 5 of the main text.

Each $Contrast\left( \phi\right)$ is fitted to ( 3 ) from the main text to retrieve a value for $r$ and $\left\langle N \right\rangle$. Hence, in total, 7 values of $r$ and $\left\langle N \right\rangle$ are retrieved. The mean and standard deviation of $r$ are 1.75 and 0.29 and that of $\left\langle N \right\rangle$ are 44 and 3, respectively. The average of the 7 fitted $Contrast\left( \phi\right)$ curves is plotted as a solid green line in Figure 5 of the main text.

# **VI. Experiment and contrast correction**

A linearly polarized gaussian beam with a wavelength of 532 nm is passed through a half wave plate followed by a zero-order vortex half wave plate (Thorlabs WPV10L-532) followed by a large angle axicon (Thorlabs AX2540-A-40.0^o^) to produce a focused CVB with $\Theta$=30^o^. The CVB state $\phi$ is controlled by rotating the initial half wave plate. This beam illuminates a colloidal dispersion of 30 nm $\times$ 100 nm TiO_2_ nanorods purchased from Nanochemazone (Catalog NCZR102-19) that is diluted in clean water to achieve a final volume fraction (volume of particles/total volume of the solution) of ~2.5$\times$10^-8^. Light scattered perpendicular to the illumination direction passes through a linear polarizer oriented along the propagation direction followed by a collimator and single mode fiber. Light coupled into the single mode fiber is split in 2 using a 50/50 fiber beam splitter and each output is connected to Hamamatsu PMTs (H7421-40). The outputs from both PMTs are connected to a time-correlated single-photon counting correlator card (TimeHarp 260, PicoQuant) which measures the intensity-intensity cross correlation: $g^{\left( 2 \right)}\left( \tau\right)-1=\frac{\left\langle I_{1}\left( 0 \right)I_{2}\left( \tau\right) \right\rangle}{\left\langle I_{1} \right\rangle\left\langle I_{2} \right\rangle}-1$, where $I_{j}$ represents the signal detected by PMT $j$ and $\left\langle\ldots\right\rangle$ represents ensemble or time averaging. Measured $g^{\left( 2 \right)}\left( \tau\right)-1$ are fitted to $a\cdot\exp\left( -\frac{\tau}{\tau_{c}} \right)+c$ and the measured contrasts (filled circle in Figure 6(c) of main text) are the values of $a+c$. Since our concentrations are very low, the measured correlograms may have a slower decay due to the number fluctuations within the interaction volume^7^. To account for this, we add a term ‘$c$’ in our correlogram fitting equation. This procedure is performed for several input states $\phi$ and the contrast is measured 3 times for each $\phi$. Typical measured and fitted correlograms for some values of $\phi$ are shown in Figure S5. Note that the measured correlogram for an azimuthally polarized illumination ($\phi=$90^o^) is noisier than that for a radially polarized illumination ($\phi=$0^o^), which is a consequence of the lower photon count rate for an azimuthally polarized illumination.


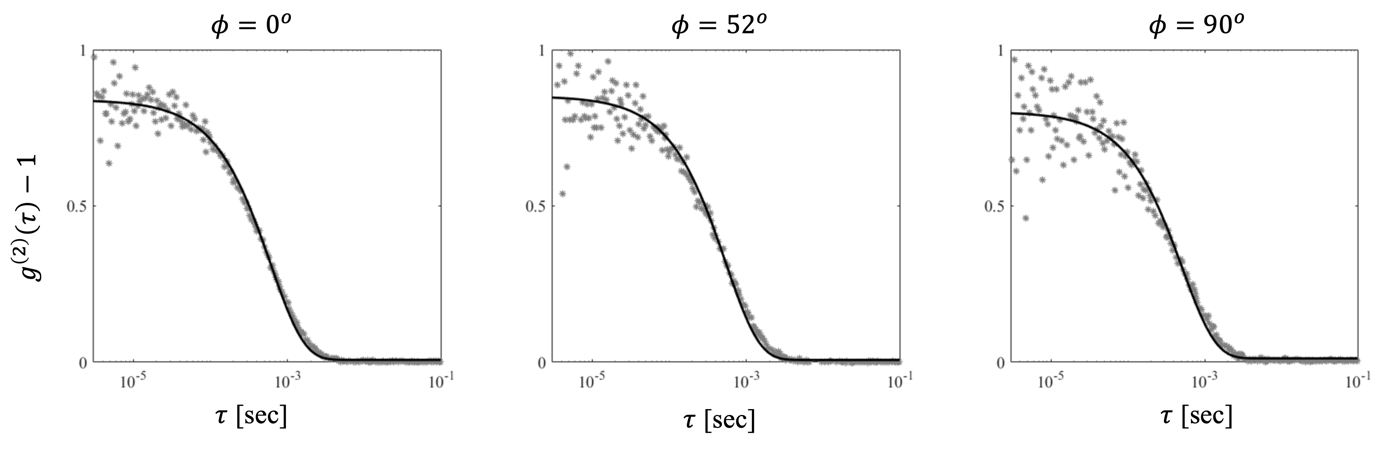


Figure S5: Typical correlograms (gray stars) measured for 3 different input states and fitted curves in the black solid line.

While, in principle, the contrast is expected to increase to a value greater than 1 as the concentration of the sample decreases, in practice, this effect is suppressed if the signal to noise ratio in the detectors is not sufficiently high. In our setup, we attribute the origin of the noise to reflections from the optics in the system, such as impurities in the glasses used and reflections from the container, and hence, it is practically unavoidable. However, if this noise can be measured, then the true contrast can be calculated by correcting the measured contrast for the noise, as shown in the following.

**Contrast Correction**

Let $E_{S,j}$ and $E_{N,j}$ represent the fields of the signal and noise measured by PMT $j$. Hence, the intensity measured by PMT $j$ will be $I_{j}=\left\langle\left| E_{S,j}+E_{N,j} \right|^{2} \right\rangle=\left\langle\left| E_{S,j} \right|^{2} \right\rangle+\left\langle\left| E_{N,j} \right|^{2} \right\rangle+2\left\langle\left| E_{S,j} \right|\left| E_{N,j} \right|\cos\left( \Phi_{S}-\Phi_{N} \right) \right\rangle$ where $\Phi_{S}$ and $\Phi_{N}$ represent the phases of signal and noise and $\left\langle\ldots\right\rangle$ represents ensemble or time averaging. Since the noise occurs due to static components (optics, container, etc.), we don’t expect it to change during the course of the measurement and, without a loss of generality, can set $\Phi_{N}=0$. Since the particles in the media are randomly diffusing over a scale much larger than the wavelength, we can also assume $\Phi_{S}$ to be uniformly distributed over the range of $0$ to $2\pi$. Since the signal and noise fields are also independent, we can write $I_{j}=I_{S,j}+I_{N,j}$ where $I_{S,j}=\left\langle\left| E_{S,j} \right|^{2} \right\rangle$ and $I_{N,j}=\left\langle\left| E_{N,j} \right|^{2} \right\rangle$. The measured correlogram can now be expressed as

$$g_{meas}^{\left( 2 \right)}\left( \tau\right)-1=\frac{\left\langle I_{1}I_{2} \right\rangle}{\left\langle I_{1} \right\rangle\left\langle I_{2} \right\rangle}-1=\frac{\left\langle\left( I_{S,1}+I_{N,1} \right)\left( I_{S,2}+I_{N,2} \right) \right\rangle}{\left\langle I_{S,1}+I_{N,1} \right\rangle\left\langle I_{S,2}+I_{N,2} \right\rangle}-1.$$

Since signals and noises in different PMTs are independent, we use $\left\langle I_{S,j}I_{N,k} \right\rangle=\left\langle I_{N,j} \right\rangle\left\langle I_{N,k} \right\rangle$ for $j\neq k$ to write the above equation as

$$g_{meas}^{\left( 2 \right)}\left( \tau\right)-1=\frac{\left\langle I_{S,1}I_{S,2} \right\rangle-\left\langle I_{S,1} \right\rangle\left\langle I_{S,2} \right\rangle}{\left\langle I_{S,1} \right\rangle\left\langle I_{S,2} \right\rangle+\left\langle I_{S,1} \right\rangle\left\langle I_{N,2} \right\rangle+\left\langle I_{N,1} \right\rangle\left\langle I_{S,2} \right\rangle+\left\langle I_{N,1} \right\rangle\left\langle I_{N,2} \right\rangle}$$

$$=\frac{\left\langle I_{S,1}I_{S,2} \right\rangle-\left\langle I_{S,1} \right\rangle\left\langle I_{S,2} \right\rangle}{\left\langle I_{S,1} \right\rangle\left\langle I_{S,2} \right\rangle\left( 1+\frac{1}{\beta_{2}}+\frac{1}{\beta_{1}}+\frac{1}{\beta_{1}\beta_{2}} \right)}$$

where $\beta_{j}=\frac{\left\langle I_{S,j} \right\rangle}{\left\langle I_{N,j} \right\rangle}.$ Let $\frac{1}{\beta}=\left( 1+\frac{1}{\beta_{2}}+\frac{1}{\beta_{1}}+\frac{1}{\beta_{1}\beta_{2}} \right)$, we can write the above as

|  | $\frac{g_{meas}^{\left( 2 \right)}\left( \tau\right)-1}{\beta}=g_{true}^{\left( 2 \right)}\left( \tau\right)-1$ | ( S11 ) |  |
| --- | --- | --- | --- |

where $g_{true}^{2}\left( \tau\right)=\frac{\left\langle I_{S,1}\left( 0 \right)I_{S,2}\left( \tau\right) \right\rangle}{\left\langle I_{S,1}\left( 0 \right) \right\rangle\langle I_{S,2}\left( \tau\right)}$. In ( S11 ), we have derived a signal to noise based correction of the measured correlogram. Since the measured contrast is the limit of $g_{meas}^{\left( 2 \right)}\left( \tau\right)-1$ as $\tau\to0$, the above equation is also an SNR based correction of the contrast.

To perform the above correction on our measured contrasts, we perform another measurement to find the noise in the system. This is done by replacing the container with our sample solution with an identical one with clean water and measuring the intensities for the values of $\phi$ over which the initial measurement was performed. From this, we calculate the SNR in the $j^{th}$ PMT as $\beta_{j}\left( \phi\right)=\frac{\left\langle I_{S,j} \right\rangle\left( \phi\right)}{\left\langle I_{N,j} \right\rangle\left( \phi\right)}=\frac{\left\langle I_{j} \right\rangle\left( \phi\right)-\left\langle I_{N,j} \right\rangle\left( \phi\right)}{\left\langle I_{N,j} \right\rangle\left( \phi\right)}$ where $\left\langle I_{j} \right\rangle\left( \phi\right)$ and $\left\langle I_{N,j} \right\rangle\left( \phi\right)$ are the time-averaged intensity and noise measured for CVB state $\phi$. The corrected contrasts are plotted as filled circles in Figure 6(c) of the main text.

# **VII. Size of interaction volume**

The “interaction volume” is the intersection of the illumination and detection angular apertures and in practice, will be soft-edged. While the exact dimensions of this volume strongly depend on the system alignment, the total volume can be approximated as one with 3D gaussian edges^8, 9^ as

$$V_{int}=V_{r}V_{z}=\int_{0}^{\infty} \left( J_{0}^{2}\left( k_{t}r \right)+J_{1}^{2}\left( k_{t}r \right) \right)\exp\left( -\frac{r^{2}}{w^{2}} \right)rdr\cdot\int_{-\infty}^{\infty} \exp\left( -\frac{z^{2}}{w^{2}} \right)dz.$$

where $w$ is the half width at $e^{-1}$ maximum of the 3D gaussian apodization and terms with $J_{0}^{2}\left( k_{t}r \right)$ and $J_{1}^{2}\left( k_{t}r \right)$ describe the co and cross-polarized interaction volumes.

$V_{z}$ is simply an infinite gaussian integral resulting in $V_{z}=\sqrt{\pi}w$. Using $\int_{0}^{\infty} J_{\chi}\left( k_{t}r \right)^{2}\exp\left( -\frac{r^{2}}{r_{0}^{2}} \right)rdr=\frac{r_{0}^{2}}{2}\exp\left( -\frac{1}{2}r_{0}^{2}k_{t}^{2} \right)I_{\chi}\left( \frac{k_{t}^{2}r_{0}^{2}}{2} \right)$ ^4^ and approximating $I_{\chi}\left( \eta^{2} \right)\approx\frac{\exp\left( \eta^{2} \right)}{\sqrt{2\pi\eta^{2}}}$ ^5^ for large $\eta^{2}$, $V_{r}\approx\frac{w}{\sqrt{\pi}k_{t}}$. Hence, the effective interaction volume can be estimated as

$$V_{int}\approx\frac{w^{2}}{k_{t}}=\frac{w^{2}\lambda}{2\pi\sin\left( \Theta\right)}.$$

We measure the half-width at $e^{-1}$ of the maximum for our single mode detection fiber-collimator setup to be $\approx$0.66 mm. With $w=0.66$ mm, $\lambda=$532 nm and $\Theta$=30^o^, our interaction volume is $\approx7.38\times{10}^{-14}$ m^3^.

Given that the sample has a number concentration (# of particles/volume) of $3.53\times{10}^{14}$ particles/m^3^, we have $\left\langle N \right\rangle\approx$ 26 particles in our interaction volume.

# **VIII. References**

1. Jakeman E. Polarization characteristics of non-Gaussian scattering by small particles. *Waves in Random Media* **5**, 427 (1995).

2. Jakeman E, Ridley KD. *Modeling fluctuations in scattered waves*. CRC Press (2006).

3. Bouchal Z, Olivík M. Non-diffractive Vector Bessel Beams. *Journal of Modern Optics* **42**, 1555-1566 (1995).

4. Bateman H, Erdélyi A. *Higher transcendental functions*. McGraw-Hill (1953).

5. Abramowitz M, Stegun IA. *Handbook of mathematical functions with formulas, graphs, and mathematical tables*. U.S. Govt. Print. Off. (1964).

6. Chandrasekhar S. Stochastic Problems in Physics and Astronomy. *Reviews of Modern Physics* **15**, 1-89 (1943).

7. Berne BJ, Pecora R. *Dynamic Light Scattering: With Applications to Chemistry, Biology, and Physics*. Dover Publications (2013).

8. Schaefer DW. Dynamics of Number Fluctuations: Motile Microorganisms. *Science* **180**, 1293-1295 (1973).

9. Chowdhury DP, Sorensen CM, Taylor TW, Merklin JF, Lester TW. Application of photon correlation spectroscopy to flowing Brownian motion systems. *Appl Opt* **23**, 4149-4154 (1984).
